# The Hidden Sexuality of *Alexandrium Minutum*: An Example of Overlooked Sex in Dinoflagellates

**DOI:** 10.1371/journal.pone.0142667

**Published:** 2015-11-23

**Authors:** Rosa I. Figueroa, Carlos Dapena, Isabel Bravo, Angeles Cuadrado

**Affiliations:** 1 Aquatic Ecology, Biology Building, Lund University, 22362 Lund, Sweden; 2 Instituto Español de Oceanografía (IEO), Subida a Radio Faro 50, 36390 Vigo, Spain; 3 Universidad de Alcalá (UAH), Dpto de Biomedicina y Biotecnologia, 28801 Alcalá de Henares, Spain; CSIR- National institute of oceanography, INDIA

## Abstract

Dinoflagellates are haploid eukaryotic microalgae in which rapid proliferation causes dense blooms, with harmful health and economic effects to humans. The proliferation mode is mainly asexual, as the sexual cycle is believed to be rare and restricted to stressful environmental conditions. However, sexuality is key to explaining the recurrence of many dinoflagellate blooms because in many species the fate of the planktonic zygotes (planozygotes) is the formation of resistant cysts in the seabed (encystment). Nevertheless, recent research has shown that individually isolated planozygotes in the lab can enter other routes besides encystment, a behavior of which the relevance has not been explored at the population level. In this study, using imaging flow cytometry, cell sorting, and Fluorescence In Situ Hybridization (FISH), we followed DNA content and nuclear changes in a population of the toxic dinoflagellate *Alexandrium minutum* that was induced to encystment. Our results first show that planozygotes behave like a population with an “encystment-independent” division cycle, which is light-controlled and follows the same Light:Dark (L:D) pattern as the cycle governing the haploid mitosis. Resting cyst formation was the fate of just a small fraction of the planozygotes formed and was restricted to a period of strongly limited nutrient conditions. The diploid-haploid turnover between L:D cycles was consistent with two-step meiosis. However, the diel and morphological division pattern of the planozygote division also suggests mitosis, which would imply that this species is not haplontic, as previously considered, but biphasic, because individuals could undergo mitotic divisions in both the sexual (diploid) and the asexual (haploid) phases. We also report incomplete genome duplication processes. Our work calls for a reconsideration of the dogma of rare sex in dinoflagellates.

## Introduction

Dinoflagellates are haploid microalgae extensively studied with respect to their worldwide occurrence, toxicity, and capacity to become ecologically dominant. These organisms form an exceptional group among eukaryotes due to the many peculiarities of their physiology [1] and enormous genome, which may be as large as 185 Gb [2] and consists of hundreds of chromosomes that lack both histones [3,4] and nucleosomes, but which are organized along a cholesteric crystal structure that ensures their maintenance in a permanently semi-condensed and visible state [5–7].

There are dozens of dinoflagellate species described to exhibit facultative sex [8,9]. Thus, under non-optimal conditions (generally related to nutrient deficiency) haploid vegetative cells differentiate into gametes, which mate and form diploid zygotes and, in turn, benthic cysts, which are better able than vegetative cells to resist stressful environmental conditions. After the cysts complete their mandatory dormancy period and if the environmental conditions are favorable for cyst germination, division of the germinated cell restores the haploid stage. However, because sexual processes in dinoflagellates are often cryptic and unpredictable, the relative importance of sexuality in their life histories is unclear. Dinoflagellates are first and foremost haploid, because reductive divisions are a characteristic of their zygotes. During these divisions, the nucleus undergoes a highly specialized process, known as nuclear cyclosis [10,11], that results in rapid chromosomal movement and is related to meiosis [12,13]. Early studies on chromosomal segregation patterns suggested that dinoflagellate meiosis is unusual [14], occurring in a single step in which homologous unreplicated chromosomes in a diploid cell form pairs that are then distributed among the haploid daughter cells. This type of meiosis differed from ordinary mitosis in a haploid cell only by the origin of the chromatid pairs that are split after metaphase. However, it was later established that meiosis in dinoflagellates generally proceeds by a more conventional two-step process but with a delay in the second division [15–18].

Nonetheless, this general model has been questioned. For example, zygotes often undergo planktonic division and thereby skip encystment. This behavior was first suggested by Uchida et al. [19] and subsequently confirmed at the nuclear level in several species belonging to the genera *Alexandrium*, *Gymnodinium*, and *Lingulodinium* (e.g., [20,21]). The haplontic model, presumed to apply to all dinoflagellates except for the diploid *Noctiluca* was recently shown to have another exception in *Polykrikos kofoidii*, which has a diplohaplontic life cycle based on evidence indicating that its zygotes either encyst and undergo meiosis (evidenced by cyclosis) or directly enter the growth cycle through what seems to be mitotic division [22]. That study supported a previous observation of mitosis in zygotes of *Cystodinium bataviense* [23].

Accordingly, either the dinoflagellate life cycle cannot be described in a single, general model or the model is more complex than originally formulated and must take into account cryptic sexuality. Sexuality is difficult to induce in the laboratory, involves complex mating patterns (e.g. [24]), and produces sexual stages that morphologically are almost identical to asexual stages (see review in [25]). Therefore, in laboratory studies the sexual interactions of dinoflagellates that occur under natural conditions may be easily overlooked (e.g. [26,27]). In fact, recent studies of blooming dinoflagellate populations showed unexpected genetic variability in populations believed to be the result of predominantly asexual, mitotic growth (e.g., [28–30]).

In this study, we followed DNA content and nuclear/chromosomal changes through the process of sexual induction and encystment in a sexual laboratory population of dinoflagellates using imaging flow cytometry, cell sorting and Fluorescence In Situ Hybridization (FISH). Our focus on one of the most well-studied dinoflagellate species, toxic bloom-forming *Alexandrium minutum*, led us to question the established dinoflagellate life cycle model, as our results clearly demonstrate that planktonic zygotes undergo an encystment-independent form of division that follows a diel and light-dependent rhythm similar to that of the haploid, mitotic cycle. This previously unknown division cycle challenges the paradigm of rare sex in *A*. *minutum* and probably in other species as well. We end our report by discussing the implications of our findings and suggest that the frequency of sex in this eukaryotic, facultatively sexual group has been greatly underestimated.

## Materials and Methods

### Ethics Statement

No activity during this study involved any endangered species or protected species.

### Culture maintenance

The dinoflagellate strains used in this study were *A*. *minutum* Mediterranean strains AL10C and VGO577, regularly maintained at the Centro Oceanográfico de Vigo (CCVIEO; Culture Collection of Harmful Microalgae of the Spanish Institute of Oceanography). AL10C and VGO577 were isolated in 2002 from Estartit (Spain) and near Girona (Spain), respectively. Both strains were cloned in 2012, resulting in clone H5 and clone H7 respectively [31]. In this study, they were cultured at a temperature of 19.5°C ±1°C with an approximate illumination of 90 μmol photons m^-2^ s^-1^ and a photoperiod of 12:12 h light:dark (L:D), with the light period starting at 08:00 h and finishing at 20:00 h. The cells were grown and maintained in L1 medium [32] without added silica. The medium was prepared with Atlantic seawater and adjusted to a salinity of 30 psu by the addition of sterile distilled water.

### Experimental setup

The experiment was carried out in two Erlenmeyer flasks, one containing a clonal culture and the other a mixture of two sexually compatible clones (cross culture). When the stock clonal cultures of H5 and H7 reached a concentration of 8,000–10,000 cells mL^-1^ (corresponding to the exponential growth phase), they were transferred to complete darkness for 66–70 h, following the method of Taroncher-Oldenburg et al. [33]. These synchronized stocks of the clonal and cross cultures served as the inoculum in subsequent experiments. In the following, we will denominate the period of time (days or hours) after the cultures are returned to the light as time post-synchronization, abbreviated as “PS”.

Clonal culture: To establish asexual growth, approximately 2000 cells of clonal strain H7 mL^-1^ were inoculated into fresh complete L1 medium. Sampling started 27hs PS after inoculation to allow cell recovery and the initiation of division. Samplings were performed every other hour during two 24-h L:D periods on days PS2 (second L:D period) and PS3 (third L:D period) to assess exponential growth in the absence of nutrient limitation, as sexuality may be induced even in nutrient-limited cultures, even if they are clonal (e.g., [21]). The culture was screened for the presence of resting cysts until day PS20.

**Fig 1:**
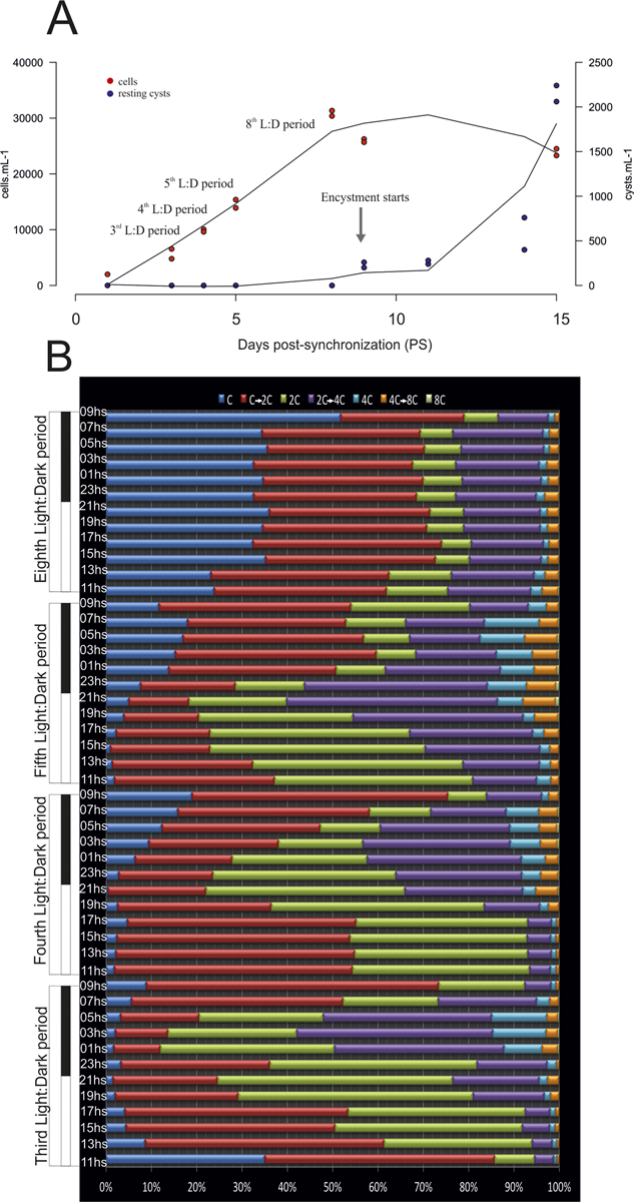
1A. Growth and resting cyst production in the cross culture. 1B. Percentage of cells in the different phases of the cell cycle during the four L:D periods studied in the cross culture.

Cross culture: Sexuality was induced by inoculating 1000 cells mL^-1^ from each clonal culture (H5 and H7) into fresh L1 medium without added phosphates. Sampling started 48hs PS to allow cell recovery and zygote formation, as reported by Figueroa et al. [34]. Samplings were performed every other hour during four 24-h L:D periods on days PS3, PS4, PS5, and PS8 (Fig 1A) by collecting one sample every other hour and starting and finishing each cycle at 09:00 h. One duplicate sample was taken on day PS15, when cyst formation had reached a maximum (cyst sample, Fig 1A).

### Sampling

An automatic water sampler (AWS, EnviroTech Instruments, Chesapeake, VA, USA) was used to collect 50-mL samples every other hour, allowing 1 min of programmed soft magnetic shaking (5 rpm, Variomag Biosystem Stirrer for Cell culture, Thermo Fisher Scientific Inc.) just before sampling. For the determination of absolute cell and resting cyst numbers, two 2-mL samples were collected manually each day, fixed with Lugol, and counted in an inverted microscope at 100× magnification using a Sedgwick-rafter chamber. At least 400 cells were counted.

### Flow cytometry analyses

#### Imaging flow cytometry (IFC)

Each 50-mL sample was filtered through a 5.0-μm-pore-size membrane filter (Millipore, Ireland), fixed with 0.5% formaldehyde for 10 min, and centrifuged at 5000 × g for 5 min. The pellet was resuspended in 2–5 mL of cold methanol and stored for at least 12 h at 4°C to facilitate chlorophyll extraction. The cells were then washed in PBS (pH 7, Sigma-Aldrich, St. Louis, MO, USA) and the pellet was resuspended in staining solution [30 μg propidium iodide (Sigma-Aldrich) mL^-1^ and 100 mg RNaseA (Sigma-Aldrich) mL^-1^ in PBS] for at least 3 h in darkness before analysis. A Flow Sight image flow cytometer (Amnis, Seattle, WA, USA) equipped with two lasers, emitting at 488 and 405 nm, was used. The samples were run at low speed and data were acquired until 15,000–50,000 events had been recorded. Ideas 6.0 (Amnis) and FlowJo 7.6 (Tree Star, Ashland, OR, USA) were used to compute peak numbers, coefficients of variation (CVs), and peak ratios for the DNA fluorescence distributions in a population. CVs were usually <7; those >10 were discarded from the analyses.

#### Cell-cycle analyses by IFC

The gradient root mean square was used to select focused cells, which have a higher gradient than unfocused cells [35]. Aggregates were eliminated based on cellular area and nuclear fluorescence. The results were verified visually by evaluating and comparing the images of discarded and retained populations. Each cell cycle phase was delimited by means of a histogram of propidium iodide fluorescence using 405-nm laser excitation, since emission at 488 nm (also recorded) was saturated even at minimum power. Cells were classified as "individual" or as in "two-cell chains" according to the nuclear-aspect ratio (width vs. height of the mask used to more precisely adjust the area to the U-shaped nucleus). The precision of the adjustment was determined manually in the control sample by analyzing the acquired images, which allowed the construction of a general template for all samples. Cell cycle control regarding the position and variability of C and 2C peaks was assessed in the clonal culture (H7) kept in darkness for 72h, without a light phase, which prohibited a shift to S phase [33,34].

Cell sorting: Cells at the different DNA content peaks (>200 cells per population) were sorted at low speed and in high purity mode in a SH800Z cell sorter (Sony Biotechnology Inc.) equipped with a 488 nm diode laser.

### Growth parameters

The specific growth rate [36] was estimated as K′ = $\frac{Ln\left(\frac{N2}{N1}\right)}{t2-t1}$, where N1 and N2 are the cell counts at times t1 and t2 of the exponential phase of growth.

Divisions per day were calculated as Div.day^-1^ = $\frac{K^{\prime }}{Ln2}$

### Optical microscopy

Cells in sorted cells and whole samples from the clonal and cross cultures were imaged at 1000× magnification (Leica DMR, Germany) using a microscope camera (Axiocam HRC, Zeiss, Germany). They were treated as described for the flow cytometry analyses except that the nuclei were stained with DAPI (2 μg mL^-1^) and the thecal plates with Calcofluor.

### Fluorescence in situ hybridization (FISH)

#### Slide preparation

The cells were harvested by gentle centrifugation at 1200 × g, treated with Liquinox as described in [37], and fixed in ethanol:acetic acid 3:1 (v/v) for at least 24 h. The fixed cells were then squashed onto clean microscope slides in a drop of 45% acetic acid. The slides were frozen, the coverslips were removed, and the sample allowed to air dry.

#### DNA probes

Plasmid pTa71 was used as the DNA probe to map the rDNA genes. It contains a 9-kb *Eco*RI fragment from *Triticum aestivum* that includes the 18S-5.8S–26S rDNA region and intergenic spacers [38]. pTa71 was labeled with digoxigenin-11-dUTP using a kit from Roche (Dig-nick translation mix).

Telomeres were detected using the deoxyribonucleotide oligomer probe (5´-CCCTAAA-3´)_3_, synthesized with Dy547 (red), at both ends (Isogen Life Science).

#### Procedure

Cell preparations from the 4^rd^ L:D and 8^th^L:D from the sexual culture and from the 1^st^ L:D from the clonal culture were incubated with DNase-free RNase A, post-fixed in freshly depolymerized 4% (w/v) paraformaldehyde, dehydrated in a graded ethanol series, and air-dried as described in [39]. The cells were then denatured by placing the slides in an incubator at 75°C for 7 min, with the temperature controlled using a programmable thermal controller (PT-100, M.J. Research) Hybridization was carried out by the addition of 30 μL of hybridization mixture (50% deionized formamide, 10% dextran sulfate, 2× SSC, and 0.33% SDS) containing 100 ng of the digoxigenated pTa71 probe and 2 pmol of the directly labeled telomeric oligonucleotide probe to each slide preparation, followed by incubation at 37°C, usually overnight. Post-hybridization washes of the slides were done in Coplin jars for 10min with 4× SSC/Tween20 at room temperature. The digoxigenin-labeled ribosomal probe was detected by incubating the slides in fluoresceinated anti-digoxigenin (Roche Applied Science) in 5% (w/v) BSA for 1 h at 37°C. No immunocytochemical procedures were required for the detection of the Dy-547 telomeric probe. The slides were rinsed for 10 min in 4× SSC/Tween20, DNA-stained with DAPI, and mounted in antifade solution (Vector Laboratories).

Microscopic analyses were conducted using an epifluorescence Axiophot Zeiss system. Images were captured with a cooled CCD camera AxioCam MRc and merged using Adobe Photoshop. The images were optimized for best contrast and brightness using the same program but only those functions that treated all pixels in the image equally.

#### Statistical analyses

Analyses were performed using the statistical and programming software R 2.1.12 (R Development Core Team, 2012), packages “ggplot2” and “scales”, available through the CRAN repository (www.r-project.org/).

## Results

### Identifying sexual processes through DNA content transitions in the cross culture, and comparison with the clonal culture

The growth and sampling times of the *A*. *minutum* cross culture is shown in Fig 1A. Growth stopped at approximately 30000 cells mL^-1^, which was reached on day PS8, one day before sexual resting cysts were detected at abundances of 50–100 cysts mL^-1^. Maximum encystment was recorded on day PS15, when resting cyst densities reached 1500–2200 resting cysts mL^-1^.

The flow cytometry histograms for the four L:D cycles performed in the cross (S1 and S2 Figs) were used to quantify the various DNA content groups according to the peaks obtained in the control, which lacked the S phase [34] and were used to determine the position of the C and 2C peaks. This quantification are shown in Fig 1B as relative percentages of cells with a given DNA content. The main DNA content of the population during the light period was positioned as S phase (C→2C). The percentages of 2C and 4C cells followed a similar pattern, characterized by maxima during the dark period and minima during the light period. However, the opposite was true for C→2C and 2C→4C cells: Minima of cells in 2C stages were coincident with maxima of cells in C→2C stages whereas maxima and minima of 4C-stage cells were nearly coincident with the maxima and minima of cells in 2C→4C stages. The highest abundances of 2C-stage cells (>50% of the population) occurred during the first three L:D periods (Fig 1B). During the third L:D period, cells in the 4C stage accounted for almost 25% of the population whereas during fourth and fifth L:D periods their abundance was lower (~15%). At day PS8, during the eighth L:D period, the cell cycle pattern that characterized the 3^rd^-5^th^ L:D periods was clearly lost in that: (i) the percentage of the population remaining in the C stage during both light and dark periods was much higher; (ii) C→2C stages accounted for a large proportion of the cells but the pattern was not that seen in the first L:D period; and (iii) cells in 2C and 4C stages also lost their previously observed pattern, with their abundances remaining constant and accounting for only 10% and 5% of the population, respectively.

**Fig 2:**
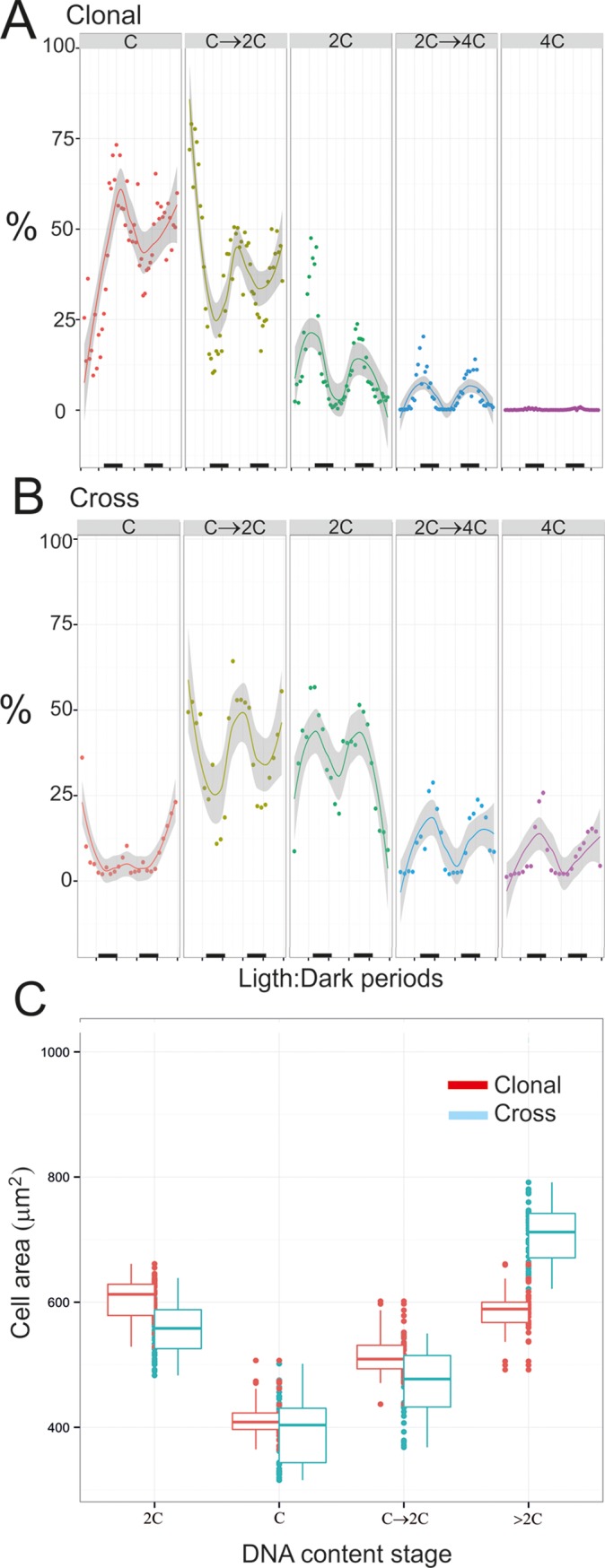
Comparisons between clonal and cross cultures. Percentage of cells in each cell cycle stage in the clonal culture (A) vs. the cross culture (B) during two L:D periods as shown by a trend line and the standard error (gray-shaded area). The dark bars along the bottom indicate the dark period. (C). Box-and-whisker plots comparing cell areas in the cell phases of clonal vs. cross culture.

The detection of important percentages of 4C stages undoubtedly point to the existence of growth through sexual processes in the cross culture, which was defined by its ability for resting cyst production due to the presence of two sexual compatible clones. However, to understand the implications of these results within a growing population, we made in Fig 2A and 2B a comparison between clonal (Fig 2A) and cross (Fig 2B) cultures for the patterns of DNA content changes during the 2^nd^ and 3^rd^ L:D periods of the clonal culture (maximum growth rate) in comparison to the 3^rd^ and 4^th^ L:D periods in the cross culture (maximum zygote formation) respectively. All DNA content stages, unless the C cells during the first L:D period, followed a similar trend in the two cultures, confirming the L:D patterns described above for the cross culture. The only prominent difference was that cells in 2C and 4C stages reached much higher abundances in the cross culture. It is interesting that the difference was much bigger for the 4C stages, given that although cells in the 2C→4C stage comprised up to 15% of the clonal culture, the transition was not completed, given that the percentage of 4C-stage cells was almost insignificant (<1%). This percentage contrasts to the value close to 25% reached in the cross culture. However, replication was not significantly completed as evidenced by the lack of 4C stages. Additionally, up to 2.3% of individual cells in the cross culture had 4C→8C DNA content, although cells with 8C DNA content were not observed. In order to further analyze the dynamics of each DNA content stage in relation to vegetative growth or sexual events, we provide in Tables 1 and 2 a summary of the maximum and minimum percentages of individual cells within a given DNA content stage and the number of cells in the cultures during each L:D period.

**Table 1:** Estimated growth parameters during sexual induction (cross culture H5×H7, phosphate-limited medium) during days 3–9 post-synchronization (DPS).

DPS	Specific growth rate (K´)	Div. day^-1^	Max (%) 2C cells^a^	Min (%) 2C cells^a^	Max (%) 4C cells^a^	Min (%) 4C cells^a^	^b^Cysts. mL^-1^
3	0.52	0.75	56.1	19.4	24.3	0.5	0
4	0.55	0.80	51.0	8.4	12.4	1.9	0
5	0.40	0.57	53.3	9.1	15.8	3.9	0
8	0.25	0.36	8.4	5.3	2.8	1	0
9	-0.16	-0.23	-	-			230±60

^**a**^Percentage values (%) only account for individual cells.

^b^The maximum number of resting cysts produced was recorded on day PS15 (1500–2200 cysts. mL^-1^).

**Table 2:** Estimated growth parameters for exponential clonal growth (culture H7, replete medium) during days PS2-3.

DPS	Specific growth rate (K´)	Div. day^-1^	Max. (%) 2C cells ^a^	Min. (%) 2C cells^a^	Max. (%) 4C cells ^a^	Min. (%) 4C cells^a^	^b^Cysts.mL^-1^
2	0.65	0.94	52.8	1.8	0.6	0.1	0
3	0.32	0.47	25.8	2.0	0.8	0	0

^**a**^Percentage values (%) only account for individual cells.

^b^No resting cysts were detected during days PS1–15.

In the cross culture (Table 1), cells in 2C and 4C stages reached their highest abundances (56 and 24%, respectively) between day PS3 and day PS5. Not all cells at the 2C stage moved to the 4C stage or returned to the C stage. Instead, a relatively large percentage (8.4–19.4%) remained in the 2C stage after the completion of each dark period, as indicated by the minimum percentage of individual 2C cells (Table 1). By contrast, practically all cells in the 4C stage disappeared from one L:D period to the next, as indicated by the minimum percentage of individual 4C cells (Table 1), which was always <4%.

During fast clonal growth, and unlike the cross culture, 2C cells practically disappeared after each dark cycle (Table 2). Although these cells had the highest rate of growth (0.94 divisions day^-1^ at day PS2) and grew twice as fast as the cross culture, the percentages of 2C and 4C cells were always lower in the clonal culture.

Following with the comparison between cross and clonal cultures, Fig 2C provides information on cellular surface during the two L:D periods compared. Differences for all DNA content stages can be seen. Although C-stage cells in the cross and clonal cultures had similar median areas, cells from the cross culture showed a greater dispersion towards smaller areas. Also, cells in C→2C and 2C stages were smaller in the cross culture. Only cells with a DNA content >2C had larger areas in the cross culture.

### Nuclear and chromosomal morphological changes: Sexual vs. asexual processes

#### IFC

**Fig 3:**
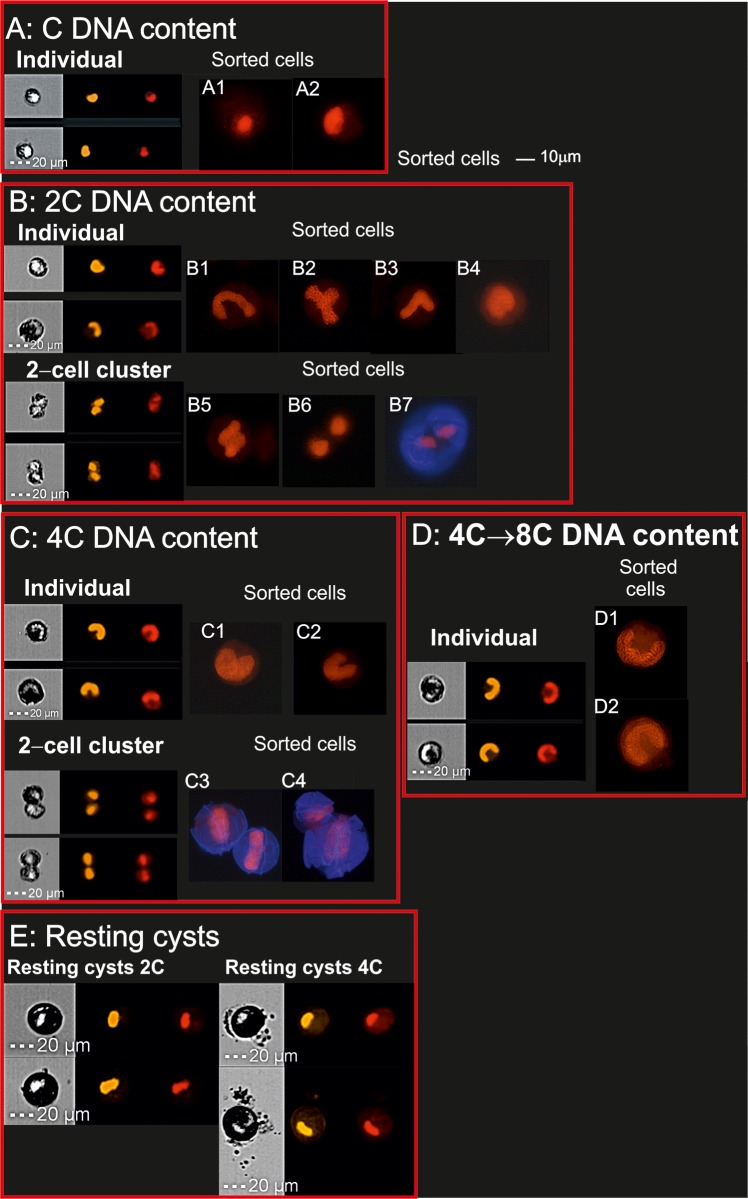
IFC and cell sorting pictures. IFC images show cell and nuclear morphologies in cells with different DNA contents as seen using bright field microscopy and after blue (488 nm, nuclei in orange) and violet (405 nm, nuclei in red) laser excitation respectively. To the right of IFC images are shown sorted cells with the same DNA content as the cells shown by the IFC pictures (A, B, D + subscript). All cells have the nuclei stained with IP. 2-clusters were additionally stained with calcofluor (in blue) in sorted cells.

**Fig 4:**
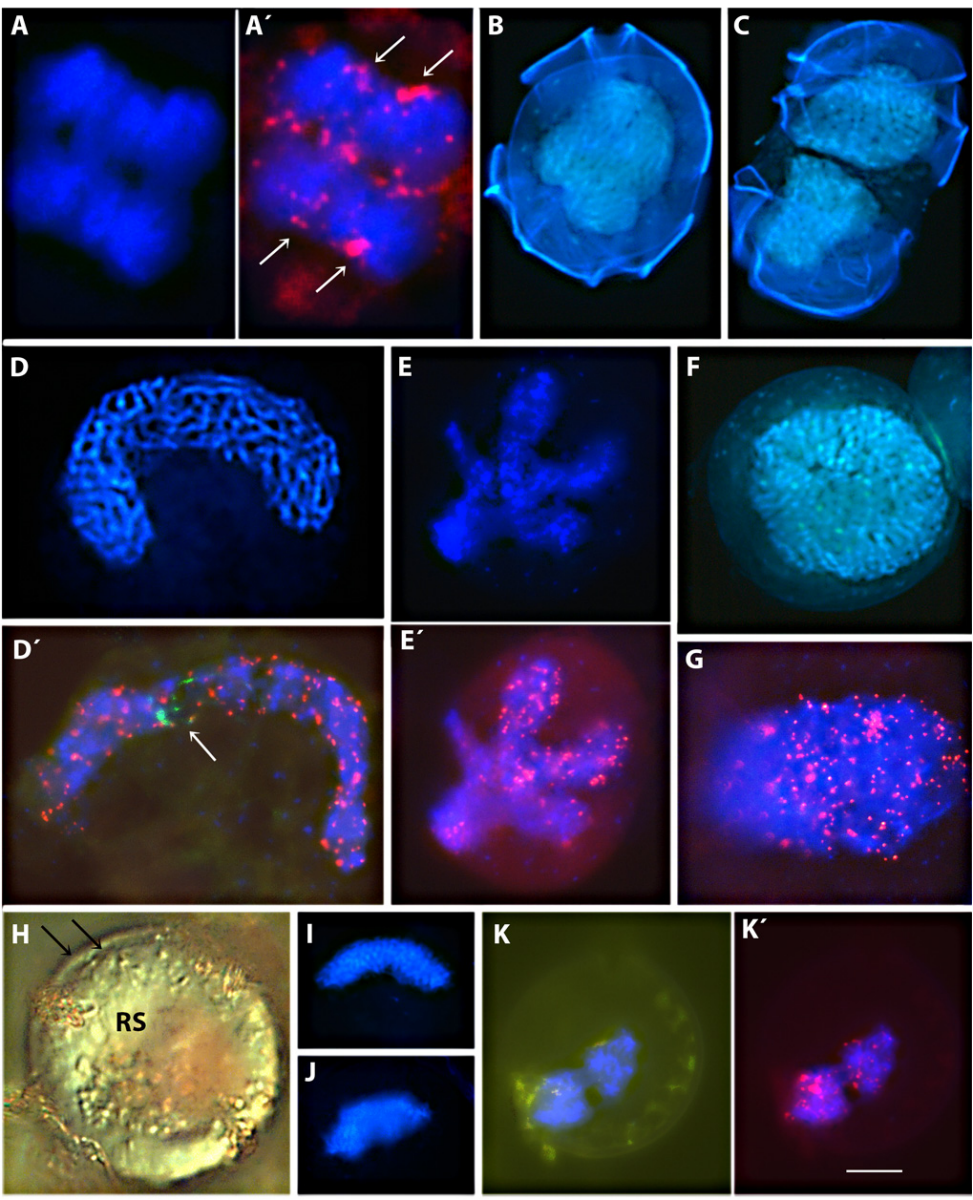
High-power magnification (1000×) images of the mitotic process in the clonal and cross cultures. DNA DAPI staining (blue) and in situ hybridization of the Dy547-labeled oligonucleotide (CCCTAAA)^3^ used to localize telomeres (red) in *Alexandrium minutum* cells. Scale bars = 10 μm

Images from the IFC and sorted cells are shown in Fig 3. The IFC images showed that C-stage cells had small, non-U-shaped nuclei (Fig 4A), although different nuclear condensation states were observed as shown between the sorted cells A1 and A2. U-shaped nuclei were typical of many 2C, 4C and higher DNA content individual cells (Fig 3B–3D and sorted cells B1, C2, D1, D2). However, other morphologies were also detected in the 2C DNA content group, in which we have summarized the other most common morphologies in three types, comprising “T”, “V” and roundish nuclei (sorted cells B2-B4). These morphologies are related to gamete fusion (“T” and “V”) and probably DNA replication (roundish nuclei, B4). 2- cell clusters belonging to the 2C DNA content category comprised different stages of vegetative division (Fig 3B lower panel, and sorted cells B5-B7), during which pairs of cells connected by a small amount of cytoplasm persisted. The IFC images and the cellular and nuclear areas allowed the discrimination of these cells from aggregates. We have used calcofluor to stain the sorted 2-cell clusters as in B7, C3 and C4, in order to show the relation between the nuclear morphology and the cytoplasmic division process, being the outer morphology of these cells typical of a cell dividing by desmoschisis. However, division by desmoschisis in 4C stages (Fig 3C, 2-cell clusters) has never been described before. Apart from their DNA content, there were found big differences in nuclear and cell size of individual and 2-cell clusters of 4C and 4C→8C stages (Fig 3C and 3D) compared with the previously described 2C stages, being observed how the U-shaped nuclei become thicker and the number of visible chromosomes is increased; notice for example the huge amount of chromosomes in Fig 3D1 and 3D2.

Encystment was only observed in the cross culture. It started at day PS9 and reached a maximum at day PS15 (Table 1). In addition to following the cells during the L:D periods studied every 2 h, we analyzed the DNA content at day PS15 in the sexual culture and detected resting cysts with 2C, 2C→4C, and 4C DNA content. Approximately 90% of the cells present in those peaks were resting cysts, identified morphologically by IFC (Fig 3E). The nuclei of resting cysts ranged from roundish to elongated but were only rarely U-shaped. The external morphologies of the cysts were the same as described by Figueroa et al. [34], either round or discoid, such that they appeared darker than the other cells.

#### FISH

Nuclear and chromosomal morphologies were examined after FISH labeling for telomeric and ribosomal sequences. The haploid mitotic process was commonly observed during the dark cycle in both cross and clonal cultures and was characterized by wide chromosomal separation, as the chromosomes were pulled towards each cell pole (Fig 4A). There was a high degree of symmetry in the distribution of telomeres during division (Fig 4A’ arrows), which occurred while the outer cellulosic thecal plates were still mostly intact (Fig 4B). Nuclear division was complete when the thecal plates were split between daughter cells (Fig 4C).

Fig 4D–4G show several processes in the cross culture directly related to the sexual cycle. Cells with nuclei as in Fig 4D–4D’ were identified as planozygotes, which have very elongated U-shaped nuclei and distinctly separated chromosomes [34]. Cells with this nuclear morphology contained only a single nucleolus (arrow in Fig 4D’). An example of gamete nuclear fusion, as described by Figueroa et al. [20], is shown in Fig 4E–4E´. This picture shows chromosomal distribution through telomeres location during gamete fusion, adding information to the “T” and “V” nuclear morphologies described in Fig 3. All these morphologies fit the variable ways in which two U-shaped nuclei have been reported to fuse, as described in [20]. Cells with large, round nuclei and strongly separated chromosomes are shown in Fig 4F and 4G, suggesting a stage of DNA replication. These cells were very abundant in the cross culture during the dark period, and were similar to the morphologies observed by IFC in some of the individual cells shown in Fig 3B, and with a higher magnification in the sorted cell in 3B4.

Resting cysts with the typical external morphology of *A*. *minutum* cysts are shown in Fig 4H–4K. In contrast to the single wall of the cells, cysts were surrounded by a thick cell wall (Fig 4H, double arrows), a red spot (RS), and an irregular and flat surface with a thick coating of mucus. The different cysts nuclear morphologies ranged from small, compact, and non-U-shaped to bilobulated (Fig 4I–4K). Clumping of the telomeric signals evidenced in these nuclei a high degree of chromosomal compaction (Fig 4K´).

## Discussion

**Fig 5:**
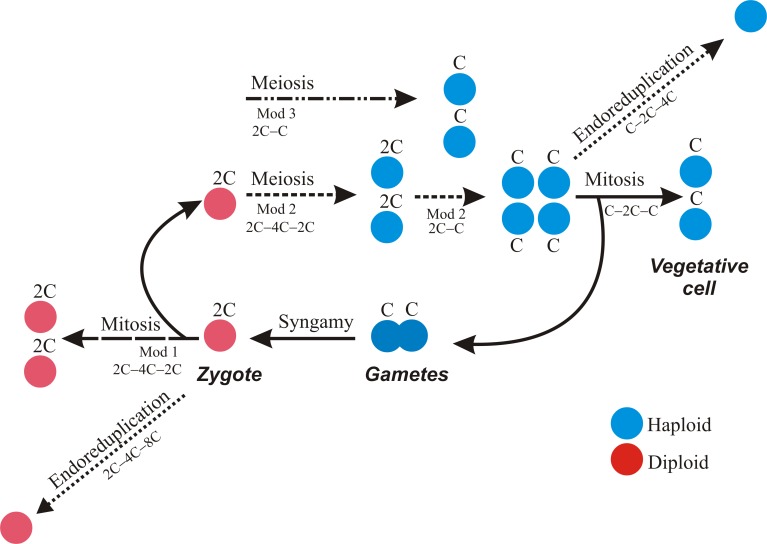
Schematic diagram of the processes that may take place in *A*. *minutum* cells during the cell cycle.

The cryptic way in which sex occurs in most dinoflagellate species poses a big stumbling block towards defining the relevance of sexuality within their life cycle. However, the main proliferative phase is considered asexual whereas the sexual phase is believed to be rare and restricted to the need to survive during adverse growth conditions through resting cyst formation. Theoretical models predict that rare sex in haploid eukaryotes can offer all the advantages of obligate sexuality at less cost, as low recombination rates could prevent genome deterioration, remove deleterious mutations and favor adaptation to drastically changing environments [40]. If this theory were true, dinoflagellates would fit this model and take profit of the advantages of sex performing it rarely and only due to the existence of very specific and stressful conditions. However, our data do not support this model and here we put forward a new one, which postulate sex within the dinoflagellate life cycle as a plastic route, more frequent than the expected from the production of resting cysts- from which is independent- and regulated by light conditions. As previously documented [41,42], sexuality ended up yielding resting cysts in our experiments when nutrients were severely depleted. However, we show that earlier to that, encystment was routinely skipped via light-controlled planozygote division, which was evidenced by the detection during the dark period of a cellular shift in DNA content from 2C to 4C, a stage that was transformed later into 2C and C stages. We are not aware of any other report of light-dependent control over zygote division in any other eukaryote. What is the nature of this division? Unlike in other dinoflagellate genera, nuclear cyclosis, indicative of the occurrence of meiosis, has yet to be observed in *A*. *minutum*. However, our data on DNA content transitions point to meiosis as one of the processes involved in the planozygote division process. To clarify this aspect and why we think that planozygotes could also have a mitotic division, we will start this discussion proposing three possible life cycle and DNA content transition models (Mod1, Mod2, and Mod3; Fig 5). In Mod1, the diploid zygote undergoes mitosis, including a transition from (2C)→(4C) of DNA replication and division through (4C)→2(2C) shifts. In Mod2, zygotes after DNA replication undergo typical two-step meiosis, with a shift in DNA content of (2C)→(4C)→2(2C)→4(C). Mod3 consists of one-step meiosis in which there is no replication of chromatids; thus, the cellular DNA content undergoes a (2C)→2(C) transition. These three possible scenarios are discussed in detail in the following.

### Meiosis vs. mitosis

That some type of meiosis occurs in dinoflagellates, and specifically in the species *A*. *minutum*, is well-established. Several reports focusing on the population genetics of blooms (e.g. [29,28]) demonstrated sexual recombination in populations resulting from the germination of resting cyst deposits. Therefore, most evidence points to the resting cyst as the meiotic stage in dinoflagellates. Our data indicate that while meiosis in resting cysts (or upon resting cyst germination) is highly likely according to previous studies, meiosis and maybe also mitosis, does also occur during the division of planozygotes in a diel cycle never reported before.

A marker of sex in dinoflagellates is the presence of 4C cells, which only occur in zygotes [13,43]. In our study, both 4C individual cells and 2-cell clusters in 4C stage were detected by IFC and conventional flow cytometry during the night period, disappearing under light conditions. Additionally, morphological evidences for planozygote formation and division in this study came from IFC and cell sorting and comprised: i) the detection of planozygote nuclei as described by Figueroa et al. [44] (Fig 4D), ii) morphologies related to gamete fusion (Figs 3B2 and 3B3 and 4E, [34]) and DNA replication in 2C cells (Figs 3B4 and 4F–4G), and 2-cell chains of 2C cells (Fig 3C, 3C3 and 3C4). Therefore, the existence of a division process in planozygotes during population growth, and as a regular daily cycle in synchronized cultures, is here for the first time proved. The next question we wanted to answer was if this division is meiosis or mitosis. Our DNA content analysis provides proof for meiosis. First, our data confirm the results of Figueroa et al. [34] for *A*. *minutum* and indicate that the remaining 2C population after each dark cycle (Min % of individual 2C cells in Tables 1 and 2) is mainly comprised by planozygotes. This value includes a relatively large percentage of cells (8.4–19.4%) which remained in the 2C stage after the completion of each dark period, although practically all cells in the 4C stage disappeared from one L:D period to the next (<4%, Table 1). This conclusion is supported by a comparison between the cross (Table 1) and clonal (Table 2) cultures, as the number of 2C cells during the light period was much higher in the former. Second, the haploid-diploid turnover indicates meiosis: Cells in 2C and 4C stages reached up to 56 and 24%, respectively, in the cross culture (Table 1). However, at day PS8, before encystment started, only 5.3 and 2.8% remained. The only way in which diploid cells could return to the haploid stage is through meiosis. In the case that zygotes were always dividing by mitosis, the resting cyst would be the only route through which the population could return to the haploid stage. Nevertheless, 4C and 2C cells progressively dropped down in the culture (Table 1), being the number of 2C cells always much lower than the expected if we consider only mitosis and that 4C cells yield 2x2C cells the next L:D cycle. Therefore, we can conclude that at least some planozygotes do meiosis while dividing and skipping encystment.

Indeed, evidence for meiosis during planozygote division was previously presented in our past study using a different *A*. *minutum* cross [34], in which we however were unable to explain why the percentage of mobile zygotes in the cross culture decreased dramatically over time days before encystment was detected, as we expected zygotes to accumulate in culture until nutrient conditions trigger their encystment [41], except by those planozygotes with a deficiency of internal nutrients, which would remain as such [42,45]. Our present results convincingly show that zygote replication and meiosis account for that dramatic decrease in the concentration of mobile zygotes in the cross culture, and therefore, both studies support the fact that sexuality does not reach its peak as previously thought when nutrient pools are exhausted and signal encystment, but rather much earlier, almost immediately after crossing (day PS3). One possible explanation for this behavior is that when nutrients are moderately rather than severely limited, rapid sexual recombination (i.e., faster than via a dormant stage) may be a better strategy for generating genetically variable offspring able to cope with the nutrient-limited environment. However, if nutrient conditions worsen, encystment may be the preferred if not the only alternative, as zygote division would be severely hampered by phosphate deficiency (e.g. [21]). This dual strategy may be especially useful in highly changing environments characterized by frequent nutrient fluctuations.

Nevertheless, we do not discard that planozygotes could divide also through mitosis. We think we have some evidences for it, as suggested by (i) the fact that division follows the same L:D pattern as haploid mitosis and (ii) the presence of double flagellated cells immediately after division. The second evidence, which has been reported several times in previous works including our own report in *A*. *minutum* (e.g. [20,34]), has an explanation that comes from the fact that diploidy in dinoflagellates has been reported to be related to the presence of two longitudinal flagella, instead of the single flagellum of haploid vegetative stages. Therefore, the reports of two biflagellated cells as the first products of mobile zygote division in several species [46,20,21,47], strongly suggest mitosis. This is because the first meiotic division is most commonly reductional, such that the first two cells should have a 2C DNA content but they are haploid, and consequently they should have one single flagellum. If the division process of mobile zygotes was mitotic, this would invalidate the established dinoflagellate life cycle model. According to Raikov [48], protists can be classified as haplonts (haploid vegetative stages, with meiosis occurring only in the zygote), diplonts (diploid vegetative stage and only the gametes are haploid), or biphasic or diplohaplontic (with alternating haploid and diploid generations both represented by vegetative, dividing forms). With the exception of *Noctiluca*, which is thought to be diplontic, all dinoflagellates examined thus far are haplonts [23]. We propose that *A*. *minutum*, and probably many other dinoflagellates with similar described mobile zygote division through biflagellated cell stages, e.g., *A*. *catenella* and *A*. *taylori* [46,20] and *G*. *catenatum* and *G*. *nolleri* [21,47], could be biphasic rather than haplontic (Mod1, Fig 5). A biphasic cycle would support the observations of Tillmann and Hoppenrath in mobile zygotes of *Polykrikos kofoidii*, who also proposed that planozygotes could divide both by meiosis and mitosis [22]. This behavior has been also described in yeasts, and for example, *S*. *pombe* zygotes undergo meiosis or mitosis depending on nutritional factors [49].

Furthermore, our data may give proof for a third cell behavior, which we hypothesize is DNA endoreduplication. DNA endoreduplication is described as a process in which chromosomes follow more than one replication cycle, but nuclei and cells do not divide [50]. This process could be evidenced in our experiment by the fact that even though the clonal culture produced 2C→4C cells, 4C cells were almost insignificant. The existence of this process is further supported by the fact that the same occurred in the clonal culture at the 4C→8C transition, given that although up to 3% of individual 4C→8C cells were observed, cells with 8C DNA content were not recorded. Endoreduplication is common in plants and animals to produce polytene or polyploid nuclei that provide a mechanism to increase the level of gene expression in response to certain physiological stresses [51]. Poliploidy has been observed before in long-established algal cultures [52], being suggested that the process could be the result of culture aging. In general, not all DNA fragments in an individual chromatid replicate to the same extent during polytenization. If local endoreplication of DNA exits, the nuclei do not reach total genome duplication. An additional explanation for the existence of 2C→4C cells in the clonal and cross cultures could be that self-mating is possible but that those zygotes do never complete its replication process due to self-sex barriers. Indeed, both processes (self-mating of gametes and endoreduplication) could co-occur.

In summary, our main and novel result is that planozygotes divide through a light-dependent and diel process, perform meiosis and maybe also mitosis, and that at both at the haploid and diploid level is observed unfinished genome duplication, which could correspond to endoreduplication. These conclusions conform a new life cycle model for dinoflagellates. In case the planozygotes also perform mitosis, we could wonder what would be the advantages and theoretical support of this new hypothetical model for dinoflagellates: A model in which they were haploids with rather frequent sex and biphasic. The condition of rare sex fits well in haplontic life cycles, as evolutionary models predict that the spread of haploidy within a population will be favored only when opportunities for genome mixing are rare. If sex were frequent, then the advantages of haploidy would be shared with those of diploidy, as the latter is less vulnerable to mutations and can compensate for those that are deleterious. Consequently, the balance would favor the diplontic cycle [53]. Evolutionary theories have been indeed incapable of incorporating a biphasic system, in which there is a balance between haploidy and diploidy. Thus, while the compensation of deleterious mutations in sexual populations favors diploidy, the purging of such mutations in less sexual populations favors haploidy. Some of the explanations behind the maintenance of a biphasic system which could be assigned to dinoflagellates, and include having a heteromorphic life cycle, in which haploid and diploid phases occupy different ecological niches [54], or living in rapidly changing environments [55]. Many dinoflagellates have both planktonic and benthic life cycle phases, the latter in the form of resting cysts, although the planktonic zygote shares its ecological niche with the vegetative stage. A biphasic life cycle in yeasts is advantageous in changing environments, in which nutrient levels determine whether haploids or diploids predominate [55]. Among unicellular protists, the plasmodial slime molds Foraminifera and some Prymnesiophycea and Myxosporidia are also biphasic ([48] and references therein).

### From gametes to encystment: Gaining insight into the process of sexuality

In the following, we focus on the steps involved in the rather cryptic process of sexuality in *A*. *minutum*. As reported by Dapena et al. [56], immediately after division the population initiates DNA synthesis such that the main peak observed under light conditions is that of S phase. Those authors also reported differences in mean cell size during different cell cycle stages that could be distinguished from one another based mainly on nuclear size, which showed the largest variation. However, haploid phase cells became highly variable in size during sexuality, which was marked by the appearance of smaller cells than in the asexual culture. These small cells were probably gametes, which in dinoflagellates are usually morphologically identical to vegetative cells except that they are smaller (see reviews by [57,58]). As some 2C stages are also the product of gamete fusion, the reduction in haploid size could explain the smaller size of the 2C-stage cells in the sexual culture. The latter finding was unexpected, because zygotes are often reported as being above average in size (e.g. [43,59]). Our results suggest that this is not the case for all species and that size is not an appropriate proxy to identify zygotes, in agreement with earlier observations of *A*. *minutum* cultures [34] and with recent observations of field samples of *A*. *fundyense* [43]. It might also be the case that dividing zygotes and encysting zygotes differ in size, as, especially in species with long dormancy periods, encystment may require the extensive accumulation of nutrient reserves.

Conversely, cells in the 4C stage were larger in the sexual culture, which could reflect their greater frequency and therefore the larger number of such cells being measured. The day before encystment started, the L:D cycle of the cell phases practically disappeared, occurring in only 10% of cells in the 2C stage and 5% of those in the 4C stage. Encystment occurred in 6–7% of the population. Given that a sex rate of 5–10% was shown to yield the same advantages as 100% sex [40], this percentage of hypothetical meiotic cells would have been sufficient. However, our results suggest that encystment is more complex than previously reported because instead of exclusively diploid cysts, we detected resting cysts with a DNA content ranging from 2C to 4C, which we interpreted as the encystment of zygotes at different stages of their division cycle. Many planktonic dinoflagellates produce benthic-resting stages that allow them to colonize a new ecological niche, survive adverse environmental conditions in the water column, and disperse over long distances. The resting cysts of *A*. *minutum*, and those of most dinoflagellate species for which a sexual cyst has been described (e.g., see the review on the *Alexandrium* genus in [60]), are believed to be sexual and diploid. Resting cysts of *A*. *minutum* have a dormancy period of 1.5 months [34], which is presumably the amount of time needed to reach the physiological maturity required for germination, assuming that the environmental conditions are favorable. Since nuclear division does not start until the cells are ready to germinate, then planozygotes must be capable of encystment at any time during their division cycle. These observations support our previous conclusion that mobile zygotes can undergo meiotic division and confirm the resting cyst as also a meiotic place. According to both the growth estimates listed in Table 2 and the detection of resting cysts with a >2C DNA content, one-step meiosis (Mod3, Fig 5) in *A*. *minutum* can almost certainly be ruled out. In case meiosis was one-step, the 4C cells would be exclusively formed by planozygotes performing mitosis.

## Conclusions

It is widely assumed that sex occurs at a constant rate in facultatively sexual species. However, the frequency of sex generally depends on the condition of the individual, a pattern found broadly across facultatively sexual prokaryotes and eukaryotes [61]. In dinoflagellate populations, sexuality, and thus the frequency of sex, is highly variable and depends on the genotypes present (e.g. [24,34]), environmental conditions (e.g. [62,63,41]), and the time frame [64]. However, in this study we were able to show that sexual processes, are light-controlled similarly to the asexual division; and that they occur in a regular daily cycle in synchronized populations. Even if the division process of the planozygote remains to be fully characterized at the cytogenetic level, our results provide strong evidence for a previously unreported plasticity and complexity of the dinoflagellate life cycle, which involves sex and meiosis as a regularly occurring phase during population growth. Some evidences suggest that other processes as planozygote mitosis and cell endoreduplication may also occur. In any case, the previously unknown complexity of the cell cycle transitions presented here changes the paradigm of rare sex in dinoflagellates and offers a new peculiarity to the already unique dinoflagellates, as to our knowledge, also opens the range of possibilities for the regulation of facultative sex in eukaryotes.

## Supporting Information

S1 Fig DNA content groups in the cross culture during 3^rd^ to 5^th^ Light:Dark periods. The control sample (in orange at the bottom of the graphs) lacked the S phase [34] and was used to determine the position of the C and 2C peaks. The DNA content of the population during the light period manifested as a single, wide peak identified as S phase (C→2C). During the dark period, it can be observed that the DNA content of the cells shifted both to the left (C stage) and to the right (2C and 4C stages) of this initial position. The presence of cells with 2C→4C and 4C DNA contents was unexpected in a population of haploid cells whose DNA content following replication should have increased from C to 2C, and then returned to the C DNA content through a round of mitotic division. Therefore, the detection of 2C→4C and 4C cells indicates that processes of planozygote formation and replication occurred from the beginning of the measurements and parallel to the regular asexual growth of the haploid population. Notice that in the fourth L:D period it is more clear the transition of the DNA content of some of the cells to the C stage based on the appearance of a defined peak to the left of the main peak, at 01:00 am.
(TIF)

S2 Fig DNA content groups in the cross culture during the 8^th^ Light:Dark period. The control sample (in orange at the bottom of the graphs) lacked the S phase [34] and was used to determine the position of the C and 2C peaks. The eighth L:D period (S2 Fig) was chosen due to the detection at day PS9 of the first resting cysts in the culture and it was characterized by a general lost of patterns for all the different DNA content stages. Contrarily to the observed in the previous cycles, the population was positioned at the C stage during the light period and there was no significant variation between light and dark periods with respect to the proportion of cells with a ≥2C DNA content; rather the percentage remained almost constant, with only a small peak.
(TIF)

## References

[1] Hackett JD, Anderson DM, Erdner DL, Bhattacharya D. Dinoflagellates: a remarkable evolutionary experiment. Am J Bot. 2004;91(10): 1523–1534. 10.3732/ajb.91.10.1523 21652307

[2] Wisecaver JH, Hackett JD. Dinoflagellate genome evolution. Annu Rev Microbiol. 2011;65: 369–387. 10.1146/annurev-micro-090110-102841 21682644

[3] Lin S, Zhuang H, Tran B, Gill J. Spliced leader-based metatranscriptomic analyses lead to recognition of hidden genomic features in dinoflagellates. Proc Natl Acad Sci. 2010;107(46): 20033–20038. 10.1073/pnas.1007246107 21041634 PMC2993343

[4] Roy S, Morse D. A full suite of histone and histone modifying genes are transcribed in the dinoflagellate *Lingulodinium* . PLoS One. 2012;7: e34340 10.1371/journal.pone.0034340 22496791 PMC3319573

[5] Bouligand Y, Norris V. Chromosome separation and segregation in dinoflagellates and bacteria may depend on liquid crystalline states. Biochimie. 2001;83: 187–192. 10.1016/S0300-9084(00)01211-6 11278068

[6] Chow M, Yan K, Bennett M, Wong J. Birefringence and DNA Condensation of Liquid Crystalline Chromosomes. Eukaryot Cell. 2010;9: 1577–1587. 10.1128/EC.00026-10 20400466 PMC2950428

[7] Rill RL, Livolant F, Aldrich HC, Davidson MW. Electron microscopy of liquid crystalline DNA: direct evidence for cholesteric-like organization of DNA in dinoflagellate chromosomes. Chromosoma. 1989;98: 280–286. 10.1007/BF00327314 2612287

[8] Pfiester LA. Dinoflagellate sexuality. In: Bourne GH, Jeon KW, Friedlander M, editors. International Review of Cytology. 1989;114: 249–272.

[9] Kremp A. Diversity of dinoflagellate life cycles: facets and implications of complex strategies In: Lewis JM, Marret F, Bradley L, editors. Biological and Geological Perspectives of Dinoflagellates. The Micropalaeontological Society, Special Publications, Geological Society, London, 2013;5: 197–205.

[10] Biecheler B. Recherches sur les Péridiniens. Bulletin Biologique de la France et de la Belgique. 1952;36 Suppl: 1–149.

[11] Pouchet G. Contribution à l'histoire des ciclioflagellés. J Anat Physiol. 1883;19: 399–455.

[12] Pfiester LA. Sexual reproduction of *Peridinium willei* (Dinophyceae). J Phycol. 1976;12: 234–238. 10.1111/j.1529-8817.1976.tb00507.x

[13] Bhaud Y, Soyer-Gobillard MO, Salmon JM. Transmission of gametic nuclei through a fertilization tube during mating in a primitive dinoflagellate, *Prorocentrum micans* Ehr. J Cell Sci. 1988;89(2): 197–206.

[14] Himes M, Beam CA. Genetic analysis in the dinoflagellate *Crypthecodinium* (*Gyrodinium*) *cohnii*: evidence for unusual meiosis. Proc Natl Acad Sci. 1975;72(11): 4546–4549. 1060138 10.1073/pnas.72.11.4546 PMC388759

[15] Von Stosch HV. La signification cytologique de la «cyclose nucléaire» dans le cycle de vie des Dinoflagellés. B Soc Bot Fr. 1972;119(sup1): 201–211. 10.1080/00378941.1972.10839089

[16] Von Stosch HV. Observations on vegetative reproduction and sexual life cycles of two freshwater dinoflagellates, *Gymnodinium pseudopalustre* Schiller and *Woloszynskia apiculata* sp. nov. Brit Phycol J. 1973;8(2): 105–134. 10.1080/00071617300650141

[17] Pfiester LA. Sexual reproduction of *Peridinum cinctum f*. *ovoplanum* (Dinophyceae). J Phycol. 1975;11(3): 259–265. 10.1111/j.1529-8817.1975.tb02776.x

[18] Pfiester LA, Timpano P, Skvarla JJ, Holt JR. Sexual reproduction and meiosis in *Peridinium inconspicuum* Lemmermann (Dinophyceae). Am J Bot. 1984; 1121–1127.

[19] Uchida T, Matsuyama Y, Yamaguchi M, Honjo T. The life cycle of *Gyrodinium instriatum* (Dinophyceae) in culture. Phycological Res. 1996;44(3): 119–123. 10.1111/j.1440-1835.1996.tb00040.x

[20] Figueroa RI, Bravo I, Garcés E. Multiple routes of sexuality in *Alexandrium taylori* (Dinophyceae) in culture. J Phycol. 2006;42: 1028–1039. 10.1111/j.1529-8817.2006.00262.x

[21] Figueroa RI, Bravo I, Garcés E, Ramilo I. Nuclear features and effect of nutrients on *Gymnodinium catenatum* (Dinophyceae) sexual stages. J Phycol. 2006;42(1): 67–77. 10.1111/j.1529-8817.2006.00181.x

[22] Tillmann U, Hoppenrath M. Life Cycle of the pseudocolonial dinoflagellate *Polykrikos kofoidii* (Gymnodiniales, Dinoflagellata). J Phycol. 2013;49(2): 298–317. 10.1111/jpy.12037 27008517

[23] Pfiester LA. Sexual reproduction In: Spector DL, editor. New York Academic Press, Dinoflagellates 1984; 181–199.

[24] Blackburn SI, Bolch CJ, Haskard KA, Hallegraeff GM. Reproductive compatibility among four global populations of the toxic dinoflagellate *Gymnodinium catenatum* (Dinophyceae). Phycologia. 2001;40(1): 78–87. 10.2216/i0031-8884-40-1-78.1

[25] Bravo I, Figueroa RI. Towards an ecological understanding of dinoflagellate cyst functions. Microorganisms. 2014;2: 11–32. 10.3390/microorganisms2010011 27694774 PMC5029505

[26] Hurst LD, Hamilton WD, Ladle RJ. Covert sex. Trends Ecol Evol. 1992;7: 144–145. 10.1016/0169-5347(92)90205-P 21235987

[27] Goodenough U, Heitman J. Origins of Eukaryotic Sexual Reproduction. Cold Spring Harb Perspect Biol. 2014;6(3): a016154 10.1101/cshperspect.a016154 24591519 PMC3949356

[28] Dia A, Guillou L, Mauger S, Bigeard E, Marie D, Valero M et al Spatiotemporal changes in the genetic diversity of harmful algal blooms caused by the toxic dinoflagellate *Alexandrium minutum* . Mol Ecol. 2013;23(3): 549–560. 10.1111/mec.12617 24330231

[29] Erdner DL, Richlen M, McCauley LA, Anderson DM. Diversity and dynamics of a widespread bloom of the toxic dinoflagellate *Alexandrium fundyense* . PloS One. 2011;6(7): e22965 10.1371/journal.pone.0022965 21829565 PMC3146535

[30] Alpermann TJ, Tillmann U, Beszteri B, Cembella AD, John U. Phenotypic variation and genotypic diversity in a planktonic population of the toxigenic marine dinoflagellate *Alexandrium tamarense* (Dinophyceae). J Phycol. 2010;46(1): 18–32. 10.1111/j.1529-8817.2009.00767.x

[31] Råberg L, Alacid E, Garces E, Figueroa R. The potential for arms race and Red Queen coevolution in a protist host–parasite system. Ecol Evol. 2014;4(24): 4775–4785. 10.1002/ece3.1314 25558368 PMC4278826

[32] Guillard RRL, Hargraves PE. *Stichochrysis immobilis* is a diatom, not a chrysophyte. Phycologia. 1993;32(3): 234–236. 10.2216/i0031-8884-32-3-234.1

[33] Taroncher-Oldenburg G, Kulis DM, Anderson DM. Toxin variability during the cell cycle of the dinoflagellate *Alexandrium fundyense* . Limnol Oceanogr. 1997;42(5): 1178–1188. 10.4319/lo.1997.42.5_part_2.1178

[34] Figueroa RI, Garcés E, Bravo I. Comparative study of the life cycles of *Alexandrium tamutum* and *Alexandrium minutum* (Gonyaulacales, Dinophyceae) in culture. J Phycol. 2007;43: 1039–1053. 10.1111/j.1529-8817.2007.00393.x

[35] Marangon I, Boggetto N, Ménard-Moyon C, Venturelli E, Béoutis ML, Péchoux C, et al Intercellular Carbon Nanotube Translocation Assessed by Flow Cytometry Imaging. Nano Lett. 2012;12(9): 4830–4837. 10.1021/nl302273p 22928721

[36] Levasseur M, Thompson PA, Harrison PJ. Physiological acclimation of marine phytoplankton to different nitrogen sources. J Phycol. 1993;29: 587–595. 10.1111/j.0022-3646.1993.00587.x

[37] Adamich M, Sweeney BM. The preparation and characterization of *Gonyaulax spheroplasts* . Planta. 1976;130(1): 1–6. 10.1007/BF00390837 24424535

[38] Gerlach WL, Bedbrook JR. Cloning and characterization of ribosomal RNA genes from wheat and barley. Nucleic Acids Res. 1979;7(7): 1869–1885. 10.1093/nar/7.7.1869 537913 PMC342353

[39] Alverca E, Cuadrado A, Jouve N, Franca S, Moreno DDLES. Telomeric DNA localization on dinoflagellate chromosomes: structural and evolutionary implications. Cytogenet Genome Res. 2007;116(3): 224–231. 10.1159/000098191 17317964

[40] D'Souza TG, Michiels NK. The costs and benefits of occasional sex: theoretical predictions and a case study. J Hered. 2010;101(suppl 1): S34–S41. 10.1093/jhered/esq005 20212007

[41] Anderson DM, Lindquist NL. Time-course measurements of phosphorous depletion and cyst formation in the dinoflagellate *Gonyaulax tamarensis* Lebour. J Exp Mar Bio Ecol. 1985;86: 1–13. 10.1016/0022-0981(85)90039-5

[42] Anderson DM. Physiology and bloom dynamics of toxic *Alexandrium* species, with emphasis on life cycle transitions. NATO ASI Ser G Ecol Sci. 1998;41: 29–48.

[43] Brosnahan ML, Farzan S, Keafer BA, Sosik HM, Olson RJ, Anderson DM Complexities of bloom dynamics in the toxic dinoflagellate *Alexandrium fundyense* revealed through DNA measurements by imaging flow cytometry coupled with species-specific rRNA probes. Deep Sea Res Part 2 Top Stud Oceanogr. 2014;103: 185–198. 10.1016/j.dsr2.2013.05.034 24891769 PMC4039218

[44] Figueroa RI, Cuadrado A, Stüken A, Rodriguez F, Fraga S. Ribosomal DNA organization patterns within the dinoflagellate genus *Alexandrium* as revealed by FISH: Life cycle and evolutionary implications. Protist. 2014;165: 343–363. 10.1016/j.protis.2014.04.001 24846057

[45] Nagai S, Yukihiko M, Oh SH, Itakura S. Effects of nutrients and temperature on encystment of the toxic dinoflagellate *Alexandrium tamarense* (Dinophyceae) isolated from Hiroshima Bay, Japan. Plank Biol Ecol. 2004;51: 103–109.

[46] Figueroa RI, Bravo I, Garcés E. Effects of nutritional factors and different parental crosses on the encystment and excystment of *Alexandrium catenella* (Dinophyceae) in culture. Phycologia. 2005;44: 658670 10.2216/0031-8884(2005)44[658:EONFAD]2.0.CO;2

[47] Figueroa RI, Rengefors K, Bravo I. Effects of parental factors and meiosis on sexual offspring of *Gymnodinium nolleri* (Dinophyceae). J Phycol. 2006;42(2): 350–362. 10.1111/j.1529-8817.2006.00191.x

[48] Raikov IB. Meiosis in protists: recent advances and persisting problems. Eur J Protistol. 1995;31(1): 1–7. 10.1016/S0932-4739(11)80349-4

[49] Davis L, Smith GR. Meiotic recombination and chromosome segregation in *Schizosaccharomyces pombe* . Proc Natl Acad Sci. 2001;98(15): 8395–8402. 10.1073/pnas.121005598 11459981 PMC37449

[50] Larkins BA, Dilkes BP, Dante RA, Coelho CM, Woo YM, Liu Y. Investigating the hows and whys of DNA endoreduplication. J Exp Bot. 2001;52(355): 183–192. 10.1093/jexbot/52.355.183 11283162

[51] Lee HO, Davidson JM, Duronio RJ. Endoreplication: polyploidy with purpose. Genes & Dev. 2009;23(21): 2461–2477. 10.1101/gad.1829209 19884253 PMC2779750

[52] Loper CL, Steidinger KA, Walker LM. A simple chromosome spread technique for unarmored dinoflagellates and implications of polyploidy in algal cultures. T Am Microsc Soc. 1980: 343–346.

[53] Mable BK, Otto SP. The evolution of life cycles with haploid and diploid phases. Bioessays. 1998;20(6): 453–462. 10.1002/(SICI)1521-1878(199806)20:6<453::AID-BIES3>3.0.CO;2-N

[54] Kondrashov AS. The asexual ploidy cycle and the origin of sex. Nature. 1994;370: 213–216. 10.1038/370213a0 8028667

[55] Gerstein AC, Otto SP. Ploidy and the causes of genomic evolution. J Hered. 2009;100(5): 571–581. 10.1093/jhered/esp057 19625454

[56] Dapena C, Bravo I, Cuadrado A, Figueroa RI. Nuclear and cell morphological changes during the cell cycle and growth of the toxic dinoflagellate *Alexandrium minutum* . Protist. 2015;166(1): 146–160. 10.1016/j.protis.2015.01.001 25681688

[57] Pfiester LA, Anderson DM. Dinoflagellate reproduction In: Taylor FJR, editor. The Biology of Dinoflagellates. Blackwell Scientific Publications, Oxford 1987; 611–648.

[58] Silva ES, Faust MA. Small cells in the life history of dinoflagellates (Dinophyceae): a review. Phycologia. 1995;34(5): 396–408. 10.2216/i0031-8884-34-5-396.1

[59] Xiaoping G, Dodge JD, Lewis J. An ultrastructural study of planozygotes and encystment of a marine dinoflagellate, *Scrippsiella* sp. Brit Phycol J. 1989;24(2): 153–165. 10.1080/00071618900650151

[60] Anderson DM, Alpermann TJ, Cembella AD, Collos Y, Masseret E, Montresor M. The globally distributed genus *Alexandrium*: Multifaceted roles in marine ecosystems and impacts on human health. Harmful Algae. 2012;14: 10–35. 10.1016/j.hal.2011.10.012 22308102 PMC3269821

[61] Bell G. The Masterpiece of Nature: The Evolution and Genetics of Sexuality California, Berkeley: University of California Press; 1982.

[62] Anderson DM, Kulis DM, Binder BJ. Sexuality and cyst formation in the dinoflagellate *Gonyaulax tamarensis*: cyst yield in batch cultures. J Phycol. 1984;20: 418–425. 10.1111/j.0022-3646.1984.00418.x

[63] Anderson DM, Coats DW, Tyler MA. Encystment of the dinoflagellate *Gyrodinium uncatenum*: temperature and nutrient effects. J Phycol. 1985;21: 200–206. 10.1111/j.0022-3646.1985.00200.x

[64] Figueroa RI, Rengefors K, Bravo I, Bensch S. From homothally to heterothally: Mating preferences and genetic variation within clones of the dinoflagellate *Gymnodinium catenatum* . Deep Sea Res Part 2 Top Stud Oceanogr. 2010;57: 190–198. 10.1016/j.dsr2.2009.09.016

