# Supplementary figures and images for: The Hidden Sexuality of Alexandrium Minutum: An Example of Overlooked Sex in Dinoflagellates
Source: PLoS One. 2015 Nov 23;10(11):e0142667. doi: 10.1371/journal.pone.0142667 (PMC4979955; doi:10.1371/journal.pone.0142667)

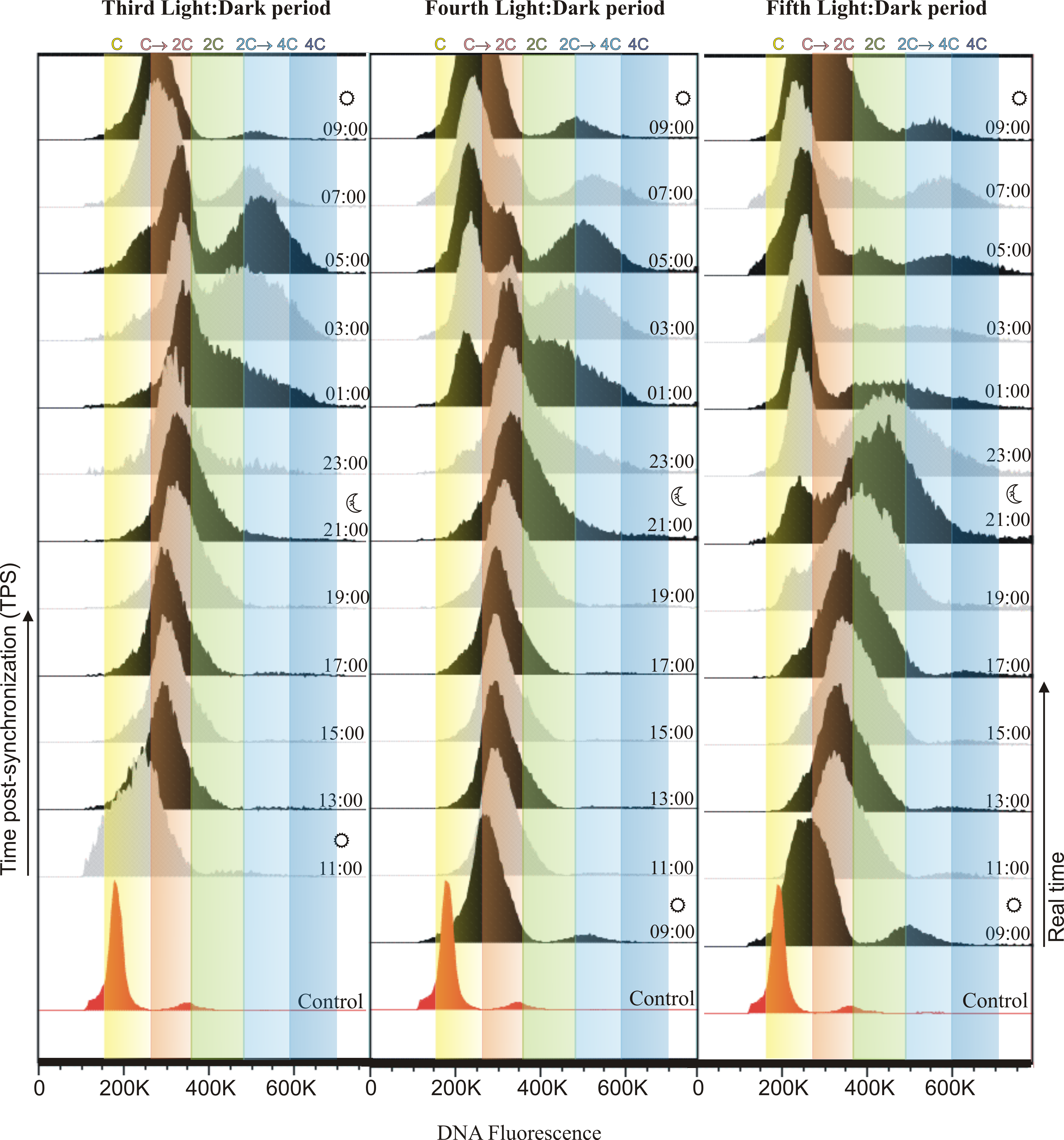

Supplement: S1 Fig — The control sample (in orange at the bottom of the graphs) lacked the S phase [34] and was used to determine the position of the C and 2C peaks. The DNA content of the population during the light period manifested as a single, wide peak identified as S phase (C→2C). During the dark period, it can be observed that the DNA content of the cells shifted both to the left (C stage) and to the right (2C and 4C stages) of this initial position. The presence of cells with 2C→4C and 4C DNA contents was unexpected in a population of haploid cells whose DNA content following replication should have increased from C to 2C, and then returned to the C DNA content through a round of mitotic division. Therefore, the detection of 2C→4C and 4C cells indicates that processes of planozygote formation and replication occurred from the beginning of the measurements and parallel to the regular asexual growth of the haploid population. Notice that in the fourth L:D period it is more clear the transition of the DNA content of some of the cells to the C stage based on the appearance of a defined peak to the left of the main peak, at 01:00 am. (TIF) [file pone.0142667.s001.tif]

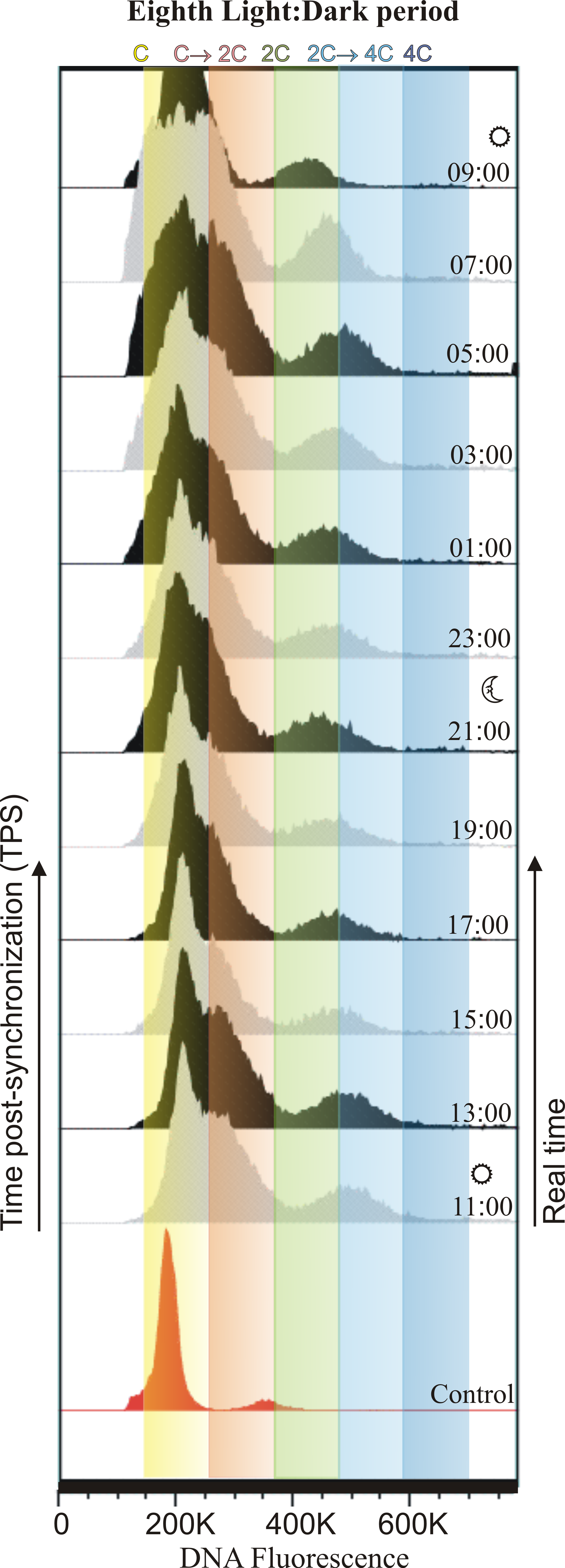

Supplement: S2 Fig — The control sample (in orange at the bottom of the graphs) lacked the S phase [34] and was used to determine the position of the C and 2C peaks. The eighth L:D period (S2 Fig) was chosen due to the detection at day PS9 of the first resting cysts in the culture and it was characterized by a general lost of patterns for all the different DNA content stages. Contrarily to the observed in the previous cycles, the population was positioned at the C stage during the light period and there was no significant variation between light and dark periods with respect to the proportion of cells with a ≥2C DNA content; rather the percentage remained almost constant, with only a small peak. (TIF) [file pone.0142667.s002.tif]
